# Analysis of the recurrence risk factors for the patients with hepatocellular carcinoma meeting University of California San Francisco criteria after curative hepatectomy

**DOI:** 10.1186/1477-7819-9-9

**Published:** 2011-01-27

**Authors:** Ruey-Shyang Soong, Ming-Chin Yu, Kun-Ming Chan, Hong-Shiue Chou, Ting-Jung Wu, Chen-Fang Lee, Tsung-Han Wu, Wei-Chen Lee

**Affiliations:** 1 Chang-Gung Transplantation Institute, Department of General Surgery, Chang-Gung Memorial Hospital, Chang-Gung University Medical School, Taipei, Taiwan

## Introduction

Hepatocellular carcinoma (HCC) is one of the most common cancers worldwide, especially in the Asia pacific area [1]. Liver transplantation is theoretically the best option because it cures both the tumor and the underlying liver disease. The overall survival rate at 5 years after liver transplantation was around 70-75%[2]. In contrast, 5-year survival rates after liver resection were only 40% to 65%, and the 10-year survival rate was 29%. The high incidence of HCC recurrence following liver resection is a serious issue. The recurrent rate is as high as 50-60% at 3 years and 70-100% at 5 years. This high recurrent rate precludes long-term tumor-free survival of the patients with liver resection for HCC. However, liver transplantation is limited by a shortage of graft availability. Liver transplantation also has high perioperative risk, and long-term problems such as graft rejection and infections[3]. Therefore, liver resection is still the primary selection treatment for many HCC patients, especially in areas lacking deceased liver.

Nevertheless, there is no doubt that for HCC, liver transplantation is a superior treatment option to liver resection, where long-term tumor-free survival is concerned. Adult-to-adult living donor liver transplantation is a well-established technique now. Liver transplantation for patients with HCC becomes feasible if a living donor wishes to donate part of the liver to save a member of the family. To optimize the benefit of living donor liver transplantation for HCC patients, the question of how to select the right patients to have liver transplantation is very important.

This study aims to identify the patients who accepted hepatectomy for a tumor/tumors and were within University of California San Francisco (UCSF) criteria[4], but had a poor 5-year disease-free survival rate (DFS). We analyze the pre-operative data of the patients and attempt to find the pre-operative risk factors of HCC recurrence. These risk factors could be indicators for clinical doctors to define and identify the patients with a high risk of tumor recurrence and to arrange liver transplantation rather than hepatectomy as the first treatment option.

## Materials and methods

### Patients

**Figure 1:**
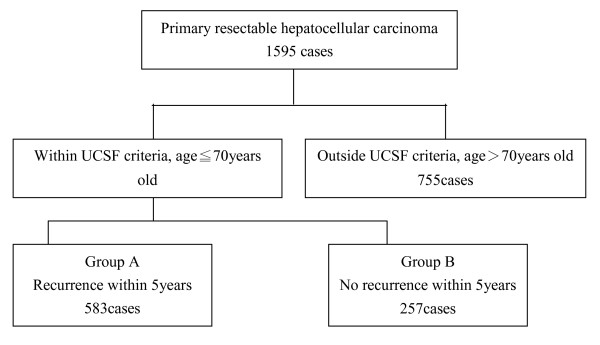
**Outcome overview of patients with resectable primary hepatocellular carcinoma (HCC) within UCSF criteria**.

A total of 1595 patients underwent hepatectomy for HCC from 1983 to 2005 in Chang Gung Medical hospital, Taipei, for whom data were collected. The patient selection criteria in this study were (1) tumor number and size within UCSF criteria, (2) no major vessel invasion, (3) no distal metastasis, and (4) age < 70 years old (based on the upper limited age of liver transplantation in HCC in this institute). Totally, 840 cases matching the criteria were the object of this study. Hospital mortality cases (expired in post-operative 30 days) were excluded from this study. Patients were further divided into two groups: group A (n = 583 (69.4%)), having tumor recurrence within 5 years after hepatectomy, and group B (n = 257 (30.6%)), showing no tumor recurrence within 5 years (Figure 1). Patient clinical data included gender, diabetes, end-stage renal disease (ESRD), smoking, and alcohol. Liver factors included HbsAg, anti-HCV, albumin, aspartate transaminase (AST), alanine transaminase (ALT), total bilirubin, alkaline phosphatase (ALK-P), alfa-fetoprotein (AFP), prothombin time (PT-INR), Child classification, and cirrhosis (detected by pre-operative liver echography). Tumor factors included size, encapsulation, vascular invasion, daughter nodule, and pathology differentiation which were recorded in pathology reports. For all the laboratory data the upper limits of normal range in our institution were chosen as the cut-off value. The cut-off values were 3.5 g/dl for albumin, 34 IU/L for AST, 36 IU/L for ALT. 94 IU/L ALK-P, 1.3 mg/dl for total bilirubin, 21 mg/dl for BUN, and 15 ng/ml for AFP.

### Recurrence

After being discharged from the hospital, patients had regular follow-up checks at 2- to 3-month intervals. Liver function was tested and alfa-fetoprotein levels were measured at every visit. Abdominal ultrasonography was used for regular follow-up visits. If ultrasonography delivered a positive finding, liver dynamic computed tomography (CT) was used to define the nature of the tumor. Recurrence was defined as the presence of radiologically confirmed tumor by CT with/without elevation of AFP. If the CT finding was controversial, hepatic angiography and liver MRI was performed to confirm the nature of the tumor.

In group A, 302 cases developed early recurrence (≦1 yr), and 281 cases were late recurrence (>1 yr). In group B (n = 287) till last following up date (2009/6/30), there were 47 patients had recurrence (16.4%), the disease free interval ranged from 60.76 to 181.78 months.

#### Statistical Analysis

The Chi-square or Fisher's Exact test was used to compare categorical variables as appropriate. Survival estimates were determined using Kaplan-Meier analysis; the results were compared by the log-rank test. Multivariate logistic regression analysis was used to identify independent factors associated with recurrence. For all statistical analysis, P < 0.05 was considered as significant. All statistical analysis was carried out using the Statistical Package for Social Science (SPSS13) for Windows.

## Result

### Outcome of hepatectomy within UCSF criteria

**Figure 2:**
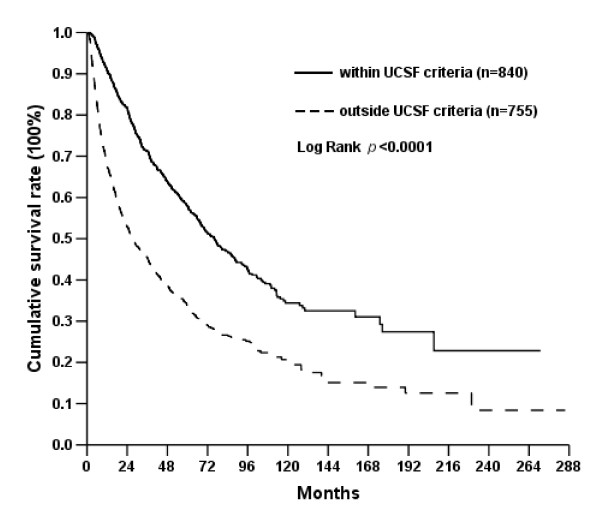
**Comparison of overall survival of patients within UCSF criteria and without UCSF criteria**.

To determine the outcome of hepatectomy for hepatocellular carcinoma, the survival rates of the patients were analyzed by Kaplan-Meier method. The 3-, 5-, and 10-year disease-free survival (DFS) rates were 39.5%, 31.2%, and 23.9%; and 3-, 5-, and 10-year overall survival (OS) rates for all the patients were 59.0%, 46.4%, and 27.7%. Hospital mortality was 5.6%. When the patients were further divided into the patients with the tumors within UCSF or beyond UCSF criteria, the 3-, 5- and 10-year overall survival rates were 71.3%, 57.9% and 34.4% for the patients with tumors within UCSF criteria which were superior to those of the patients with tumors beyond UCSF criteria (Figure 2). However, these treatment results of the patients with tumors within UCSF criteria were inferior to those who had liver transplantation reported in the literature.

#### Predictive factors for recurrence

For the patients having tumor recurrence within 5 years after hepatectomy, liver transplantation might be beneficial. To determine the risk factors of tumor recurrence, the characteristics of group A and B patients were compared. Characteristics and comparison of the two populations are listed in Table 1. According to univariate analysis, the favor factors related to a 5-year disease-free survival rate were female gender, AST < 34IU/L, ALT < 36IU/L, ALP < 94IU/L, ALB > 3.5 g/dl, AFP ≦ 15 ng/ml, no surgical complication, no cirrhosis, small tumor size.

**Table 1:** Characteristics and Comparison of the two population study

	DFS < 5 years	DFS ≧ 5 years	*P *value
Gender			0.034
Male	469 (80.4%)	190 (74%)	
Female	114 (19.6%)	67 (26%)	
Age			0.220
**≤**65	484 (83%)	222 (86.4%)	
>65	99 (17%)	35 (13.6%)	
Diabetes Mellitus			0.280
No	493 (84.7%)	225 (87.5%)	
Yes	89 (15.3%)	32 (12.5%)	
End-stage renal disease			0.360
No	567 (97.4%)	253 (98.4%)	
Yes	15 (2.6%)	4 (1.6%)	
Smoking			0.256
No	317 (58.6%)	151 (62.9%)	
Yes	224 (41.4%)	89 (37.1%)	
Alcohol			0.088
No	318 (64.8%)	152 (71.4%)	
Yes	173 (35.2%)	61 (28.6%)	
HbsAg			0.546
(-)	168 (30.9%)	80 (33.1%)	
(+)	376 (69.1%)	162 (66.9%)	
Anti-HCV			0.492
(-)	263 (57.7%)	133 (60.5%)	
(+)	193 (42.3%)	87 (39.5%)	
AST			<0.001
**≤**34	201 (35.6%)	123 (50.4%)	
>34	363 (64.4%)	121 (49.6%)	
ALT			<0.001
**≤**36	192 (35.2%)	123 (50%)	
>36	354 (64.8%)	123 (50%)	
ALK-P			0.001
**≤**94	364 (68.2%)	186 (80.2%)	
>94	170 (31.8%)	46 (19.8%)	
Albumin			<0.001
**≤**3.5	100 (18.6%)	13 (5.5%)	
>3.5	439 (81.4%)	222 (94.5%)	
AFP			0.002
**≤**15	123 (24.6%)	112 (44.8%)	
>15	378 (75.4%)	138 (55.2%)	
Bilirubin Total			0.373
**≤**1.3	480 (83.9%)	221 (86.3%)	
>1.3	92 (16.1%)	35 (13.7%)	
Bun			0.482
**≤**21	463 (86.2%)	200 (88.12%)	
>21	74 (13.8%)	27 (11.9%)	
			
PT INR			0.146
**≤**1.5	389 (97.5%)	160 (99.4%)	
>1.5	10 (2.5%)	1 (0.6%)	
Child stage			0.031
A	528 (91.7%)	247 (96.5%)	
B	44 (7.6%)	9 (3.5%)	
C	4 (0.7%)	0 (0%)	
Complication			0.04
No	459 (78.7%)	218 (84.8%)	
Yes	124 (21.3%)	39 (15.2%)	
Pathology factor			
Capsule			0.836
No	150 (28.1%)	69 (28.9%)	
Yes	383 (71.9%)	170 (71.1%)	
Daughter Nodules			0.071
No	426 (87.5%)	208 (90.4%)	
Yes	61 (12.5%)	22 (9.6%)	
Cirrhosis			0.007
No	188 (33.3%)	108 (43%)	
Yes	377 (66.7%)	143 (57%)	
Tumor size			0.013
**≤**5 cm	471 (82.3%)	228 (88.7%)	
>5 cm	104 (17.7%)	29 (11.3%)	

#### Pre-operative independent factors to predict tumor recurrence

In this study, we only focused on pre-operative detectable factors which helped to make a decision between hepatectomy and liver transplantation. By multivariate analysis male, AST > 34IU/L, albumin ≦ 3.5 g/dl, AFP > 15 ng/dl, tumor size > 5 cm in diameter were independent factors contributing to tumor recurrence within 5 years (Table 2). The output from a statistical package was given. The Wald tests showed that all 5 explanatory variables gender, AST, ALB, AFP, and tumor size contributed significantly to the model. The male-to-female odds ratio is 2.079, AST > 34 to AST ≦ 34 is 1.704, ALB ≦ 3.5 to ALB > 3.5 is 3.436, AFP > 15 to AFP ≦ 15 is 1.726, and tumor > 5 cm to ≦ 5 cm is 1.793.

**Table 2:** independent risk factors in logstic regression

Factors	Odds ratio	95% CI of odds ratio		*P *value
Gender				0.001
Male/female	2.079	1.350	3.195	
AST				0.003
>34/≤34	1.704	1.194	2.434	
ALT				0.478
Albumin				<0.0001
≤3.5/>3.5	3.436	1.739	6.757	
Alk-P				0.090
AFP				0.003
>15/≤15	1.726	1.202	2.479	
Cirrhosis				0.176
Tumor size				0.028
>5/≤5	1.794	1.064	3.021	

CI: confidence interval

**Figure 3:**
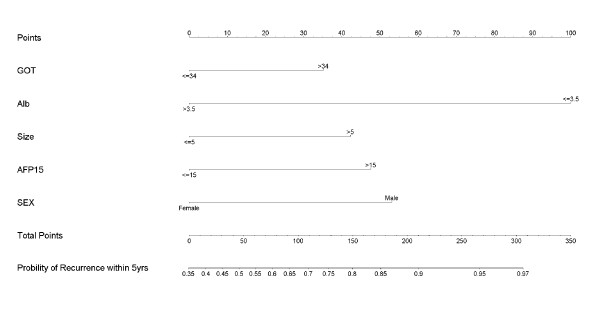
**The nomogram is to predict the probability of recurrence within 5 years after curative hepatectomy**. Instructions to using this nomogram: Locate the patient's GOT data on the axis, either >34, or ≦34. Draw a line up to the Points axis. The point is 35(if GOT > 34) and 0(if GOT ≦ 34).Repeat this process for the other predictors axes. Sum up the points for each predictor and locate the number on the Total Points axis. Draw a line straight down to the Predicted probability of recurrence with 5 years to determine the patient's probability of recurrence risk.

On the basis of our multivariate analysis of five recurrent risk factors, a nomogram was developed to predict the risk of recurrence in 5 years (Figure 3). By applying this nomogram to an individual patient's pre-operation variables, the numbers of points from each factor were cumulated to produce a total number of points for that patient. A vertical line is then drawn from the line indicating the total number of points to the line indicating the probability of recurrence in 5 years after hepatectomy.

## Discussion

The only therapies which are capable of providing cure for hepatocellular carcinoma patients are hepatic resection and liver transplantation. Despite the lack of a high-grade evidence base for either resection or transplantation, the result of these treatments provides 5-year survival rates of up to 70% in selected patients. These are clearly superior to the natural course of the disease [5-7]. Liver transplantation is theoretically the best resolution of HCC within UCSF criteria. Compared with patients under UCSF criteria undergoing liver transplantation, whose 5-year survival was 75%[8], our data revealed a 5-year survival rate of 56.8% for patients who underwent hepatectomy under the same criteria. The result is thus worse than for transplantation.

However, due to the problems of graft shortage, long waiting time, higher perioperative risk and long-term immunosupression, hepatectomy produces a considerable overall survival benefit for these patients[9]. The major benefit of hepatic resection is that it can be performed without a waiting time. Furthermore, operative outcomes after hepatic resection have improved over recent decades in cirrhotic patients. The hospital mortality rate in experienced medical centers was less than 5% in selected patients[10]. Similar to other series, we achieved a surgical mortality rate of 5.6%. Although hepatic resection is a safe therapeutic choice, the concern of the choice of hepatectomy in this group of patients is a higher risk of recurrence than transplantation.

The aims of this study were to find the recurrence risk factors for those patients with a tumor/tumors meeting UCSF criteria. This finding could assist both surgeon and patients in deciding whether they should immediately adopt primary liver transplantation. Those patients who did not have risk factors of recurrence could accept hepatectomy as their primary treatment. This may alleviate demand for liver graft or liver transplantation from living relatives. Furthermore, liver graft can be offered to high-risk group patients, so they don't need to explore salvage transplantation or drop out from the waiting list if they suffer recurrence or liver function deterioration. However, there has been some controversy regarding whether primary transplantation or salvage liver transplantation is the optimal treatment[11]. Adam et al reported that primary liver transplantation is superior to salvage liver transplantation. Secondary liver transplantation has a poorer outcome, including higher mortality and morbidity, and higher recurrence than primary liver transplantation. However, Del Gaudio M. et al mentioned liver resection had a similar 5-year overall survival to primary liver transplantation under intention-to-treat analysis, although with an increased risk of recurrence in small HCC and well-compensated cirrhosis. Salvage transplantation is still a safe and effective approach in recurrent group[12].

Our data showed that liver function reserve was a key factor of early recurrence. AST > 34IU/L and albumin ≦ 3.5 were two important risk factors of recurrence. Chronic hepatitis is a key factor of liver cell mutation resulting in malignancy. Chronic hepatitis also influenced recurrence after hepatectomy[13]. It has been reported that early tumor recurrence was related to cirrhosis, chronic active hepatitis and HCV positivity[14]. In a large-scale multivariate analysis, the risk factors and outcome of early recurrence after resection of HCC included cirrhosis, hepatitis B/C, Child-Pugh score, transaminase level, albumin level, chronic active hepatitis[5]. Patients with an elevated ALT level (>2× normal) had a risk not only of tumor recurrence, but also had a significantly higher risk of developing ascites and liver insufficiency[15]. Therefore, primary liver transplantation for HCC patient with abnormal liver function may have clinical benefit[16].

Our data also showed that pre-operative AFP > 15 was an independent risk factor of recurrence. It has been reported that AFP was an independent prognostic indicator of overall survival and disease-free survival [10,17,18]. Vibert et al. mentioned that increasing AFP > 15 ng/ml/month while waiting for LT was the most relevant pre-operative prognostic factor for low overall and disease-free survival. AFP progression could be a pre-operative marker of tumor aggression [19]. Therefore, primary liver transplantation may be considered for patients having AFP > 15 ng/ml. However, patients with both tumors >5 cm and serum AFP levels >1000 ng/mL had an 82% incidence of vascular invasion. Sakata, Shirai et al also reported that tumor sizes and serum AFP level, alone or in combination, were useful in predicting the presence or absence of vascular invasion before hepatectomy for HCC [20]. Because vascular invasion was a poor prognostic factor of tumor recurrence both for hepatectomy and liver transplantation, liver transplantation for the patients with tumor size > 5 cm and marked elevation of AFP should be highly selected.

In almost all populations, HCC male/female prevalence ratio averaging between 2:1 and 4:1 was reported[21]. A comprehensive review of literature revealed shortcomings associated with estrogen receptor (ER) and androgen receptor (AR) play an important role in normal liver and HCC[22]. In our institution, male-to-female ratio is 3.6:1. The striking gender disparity is also the risk factor of recurrence after hepatectomy. Primary liver transplantation should be a treatment option for male patients.

In conclusion, hepatectomy or liver transplantation for HCC within UCSF criteria in deceased liver donor shortage areas is a difficult decision issue. In this study, male, AST > 34IU/L, albumin ≦ 3.5 g/dl, AFP > 15 ng/ml, tumor size >5 cm in diameter were the risk factors of tumor recurrence. For the patients without or with limited risk factors of tumor recurrence, hepatectomy rather than liver transplantation will be the first choice of treatment. For the patients with risk factors of tumor recurrence, primary liver transplantation rather than hepatectomy might be the option of treatment to achieve long-term disease-free survival although tumor recurrence can not be prevented completely.

## Competing interests

The authors declare that they have no competing interests.

## Authors' contributions

RS Soong participated in the sequence alignment and drafted the manuscript. CF Lee, TJ Wu, and KM Chan participated in the sequence alignment. MC Yu, TH Wu and HS Chou participated in the design of the study and performed the statistical analysis. WC Lee conceived of the study, and participated in its design and coordination and helped to draft the manuscript. All authors read and approved the final manuscript.
